# Compound Formation in Language Mixing

**DOI:** 10.3389/fpsyg.2020.01021

**Published:** 2020-06-03

**Authors:** Artemis Alexiadou

**Affiliations:** ^1^Institute of English and American Studies, English Linguistics, Humboldt-Universität zu Berlin, Berlin, Germany; ^2^Research Area: Syntax and Lexicon, Leibniz-Zentrum Allgemeine Sprachwissenschaft, Berlin, Germany

**Keywords:** language mixing, distributed morphology, compounds, words, stems

## Abstract

In this paper, I discuss nominal compound formation in language contact situations, the question being of how compounding in language mixing can inform both theories of mixing and theories of word-hood. This contributes to our further understanding of how word formation operates in cases of language mixing and what exactly is being mixed in mixing, i.e., words vs. units smaller than words, e.g., stems or roots. Compounding is important to answer this question, as languages differ with respect to the units they employ for compound formation, i.e., phrases vs. stems. The data to be discussed will be a mixture of materials that have already been published in the literature and newly collected data and involve several mixing varieties, namely, Greek–English, Greek–Italian, Greek–Turkish, Turkish–Norwegian, Turkish–Dutch, and French–Dutch. I then offer an analysis using the tools of syntactic models of word formation (e.g., distributed morphology), assuming a decompositional approach.

## Introduction

A lot of work on language mixing aims to offer a typology of the possible mixing patterns that can be identified across language contact pairs; (see for instance Muysken, 2000; Alexiadou and Lohndal, 2018) for a recent summary. As Alexiadou and Lohndal point out, while most of this work is devoted to the study of units beyond the word level, there is a growing interest in the study of word internal language mixing, the aim being to identify the basic units that may be mixed as well as the ways in which languages vary. Alexiadou and Lohndal (2018) discuss several word-internal mixing pairs by looking at different bilingual varieties. As there are many cases where a root from one language combines with functional morphology from another, they conclude that word internal mixing is in general possible. Where such combinations violate morpho-phonological constraints, the mixings are dis-preferred. Moreover, bilingual speakers seem to prefer to make use of the functional morphology of the language that has overt realization of a particular grammatical category, which then acts as the matrix language in the sense of Myers-Scotton (1993)^1^.

Examples of word internal mixing discussed in Alexiadou and Lohndal (2018) are as in (1), an example of Greek–German mixing, and in (2), cases of Greek–English mixing, from Gardner-Chloros (2009), p. 50), next to their Greek, German, and English counterparts:

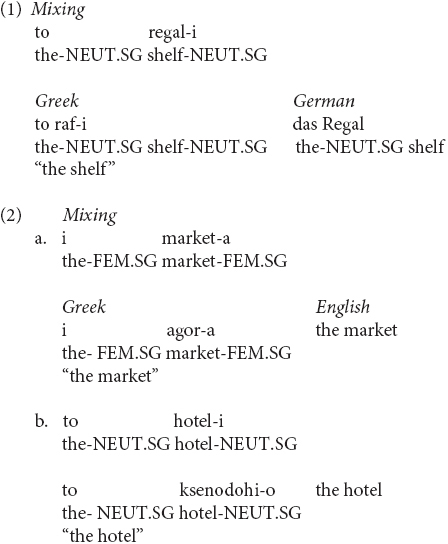



In (1–2), forms that correspond to German and English nouns combine with Greek inflectional affixes, which assign the novel nouns to one the eight declension classes available in Greek (see e.g., Ralli, 2000; Alexiadou and Müller, 2008; Alexiadou, 2017) for an analysis of Greek declension classes (DCs). For this to happen, the English nouns have to be re-analyzed as stems/roots (see footnote 6). English nouns lack inflectional information, and the combination of an English root with a Greek affix leads to DC and gender assignment, (2). German nouns also belong to several DCs (Alexiadou and Müller, 2008); nevertheless, the inflectional endings seen in (1) come from Greek^2^. Examples of this type suggest that bilingual speakers treat the German–English words as stems to which they can apply the DC information that characterizes Greek nouns. In other words, the mixing is across morpheme boundaries and the bilingual grammar treats the German and English nouns as elements without any inflectional information, i.e., as stems. Such cases are systematic and have been discussed for both the verbal and nominal domains for different contact varieties of Greek; see the references cited in Alexiadou and Lohndal (2018), and also Seaman (1972); Ralli et al. (2015), and Alvanoudi (2019) for further mixing examples involving Greek, Poplack (1980); Sankoff and Poplack (1981), and more recently López (2018) and López et al. (2017) for many different contact varieties.

Cross-linguistically, it is well-known that such mixings are asymmetric: the examples in (1–2) involve Germanic stems that combine with Greek inflectional affixes, but the reverse is not attested (see footnote 1). By contrast, in the Spanish–German variety described by González-Vilbazo (2005) and spoken in Barcelona, a German affix with DC information can attach to a Spanish stem, but the reverse is not attested, e.g., *^∗^Stuhl-o* “chair_DC_” vs. *Segerat-en_*DC*_* “security men.” In the case of (1–2), Greek is the language that provides the basic frame, i.e., it is the matrix language in the sense of Myers-Scotton (1993); in the case of German–Spanish, it is German that is the matrix language^3^.

Typically, cases of word internal mixing involve a process of affixation via which an e.g., German/English word becomes Greek, as in (1–2). The result of these combinations is that basic word units of the Greek contact variety vocabulary. In this paper, I will be concerned with multi-unit words, specifically mixed nominal compounds. Mixed nominal compounds have been discussed in the literature to some extent. For instance, Muysken (2000) cites mixed compounds as an example of the process of *congruent lexicalization*. In his discussion of the German–English mixed nominal compounds described by Clyne (1967), e.g., *beachhäuser* “beach houses” and *Kettenstore* “chain store,” Muysken (2000, p. 150) notes that the bidirectionality of the process suggests congruent lexicalization. The compounding rules are very similar in the two languages; thus, it is possible to have mixed compounds with either a German or an English head. While it might very well be that German-headed compounds are predominant, suggesting that German is the matrix language of the German–English bilingual speakers investigated by Clyne, both German-headed and English-headed compounds are possible^4^. For Muysken, mixed compounds are word-internal phenomena that are the result of a shared word grammar. The concept of a shared word grammar has been widely discussed in the literature on language mixing from a variety of perspectives: the basic question is whether bilingual speakers have one integrated lexicon or two separate lexicons (see Alexiadou and Lohndal, 2018; Putnam et al., 2018) for a recent overview, and (Sankoff and Poplack, 1981; MacSwan, 1999), and the contributions in Stell and Yakpo (2015); López (2018), and Riksem et al. (2019) for a variety of theoretical perspectives.

Mixed nominal compounds are highly interesting both for work on the interface between syntax and morphology and for work on language mixing. Furthermore, compounds offer a very fruitful domain to test theories of language processing and the mental lexicon in bilingual speakers. It has been suggested that these speakers generally have generally problems with retrieving words (see, e.g., González-Alonso et al., 2016, for a recent overview); thus, it is not clear what we expect them to do while building compounds, especially if the two languages they have at their disposal make use of distinct rules.

This study aims to answer the following questions: to the extent that bilingual speakers build mixed compounds, do we find the same asymmetries in compound formation as we do in word internal mixing, i.e., the head element may only come from one of the two languages, and may this vary across contact varieties? Do such speakers only form mixed compounds, or may they also build un-adapted ones? Finally, as compounds are internally complex, how can they inform theories of the lexicalization of concepts across language pairs? If Kroll and Stewart (1994) are correct in assuming that languages share underlying concepts, it is possible that these may be lexicalized via compounds in some languages but not in others.

I will show that indeed mixed compounds are asymmetrical and across contact varieties speakers may produce both mixed and un-adapted compounds. This suggests that speakers have two sets of rules for compound formation: one set is also available to monolinguals, and a second set is determined by one of their languages, which functions as the matrix language. In the latter case, they choose to insert material from language A to a context otherwise determined by language B. Finally, the Greek contact varieties provide evidence that while certain concepts are expressed via compounding in e.g., English, the mixed Greek–English variety makes use of a derivational process via affixation. In sum, the study of compounds in mixing will inform our understanding of the units of mixing as well as of the rules bilingual speakers have at their disposal in order to build words and phrases and how these differ, if at all, from the monolingual grammar rules.

The paper is structured as follows. In section “Rules of Nominal Compound Formation Across Languages,” I discuss the typological variation found in compound formation. I will limit the discussion to compound formation in the languages that will constitute the empirical basis of this paper. In section “Materials and Methods,” I discuss the methods of the collection of the (novel) data discussed in this paper. In section “Mixed Compounds in Language Contact,” I offer a discussion of several pairs of mixed compounds. In section “Units and Structures of Mixed Compounds,” I turn to an analysis of the patterns. In section “Conclusion,” I conclude and offer some directions for future work.

## Rules of Nominal Compound Formation Across Languages

Following Ralli (2013a, p. 183), I assume that compounds can be distinguished into two types: stem-based and word-based objects bearing an atomic status, depending on the language one deals with^5^. A simple example that Ralli (2013a, p. 185) offers to illustrate this distinction is the following: in English, the compound *tablecloth* consists of two independent words, namely, *table* and *cloth*. By contrast, in Greek *trapezomándilo* “tablecloth” involves the stems of the words *trapéz(-i)* “table” and *mandíl(-i)* “scarf, cloth” (3):

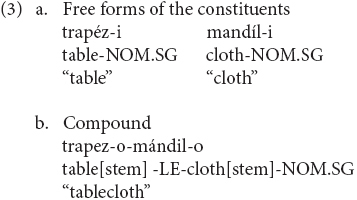



While in English the elements that are involved in compounding are fully inflected words and thus qualify as phrasal – (see also Iordãchioaia et al., 2017; Alexiadou, 2019) for further discussion – the elements of the compound illustrated in (3b) are not, since they do not appear with the DC information they are associated with when they occur in isolation, (3a).

In fact, (3b) is one of four types of compounds that Greek has, illustrated in (4), Ralli (2013b); (4a), which is the same type of example as (3b), and (4b) are stem based, while (4c–d) are phrasal, i.e., word–word compounds generally considered the result of syntactic phrasal formation; see also Gavriilidou (2013) who uses the label NN combinations. (3b), (4a), and (4b) are right headed, while (4c–d) are left headed:

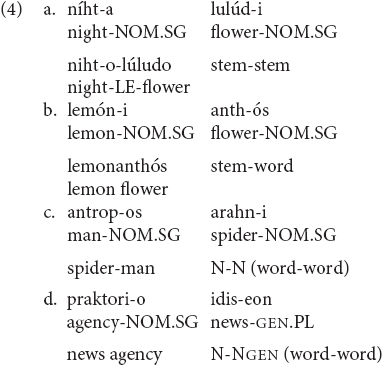



Ralli calls (3b/4a)-type compounds stem–stem compounds, while (4b) involves stem–word compounds. She makes this distinction on the basis of the following criteria. In (3b/4a), both the head and the non-head are stems^6^. In (4b), the non-head is obligatorily a stem, while the head of the compound bears the same set of inflectional affixes that it would have in isolation and thus qualifies as a word. Importantly, the compounds in (4a) and (4b) form a single stress domain. Absence of word internal inflection or derivational morphology qualifies the non-heads of these two compounds as stems, i.e., non-heads are morpho-syntactically dependent. Because of this, Ralli (2013a, b) formulates the bare stem constraint for Greek, according to which the non-heads of right-headed compounds as in (4a–b) have to be as bare as possible, i.e., derivational or inflectional affixes are not permitted word-internally.

While in (4a–b) the non-head of the compound does not a have a word status, since it is not associated with its canonical inflectional endings, the situation is different in (4c) and (4d). These compounds have two stress domains and are fully inflected words. In (4c), the two nouns are fully inflected and bear an unmarked nominative, while in (4d) the non-head bears a genitive case. Thus, an important criterion to determine the word/phrasal as opposed to the stem status of the constituents of a compound is the presence of inflectional and/or derivational morphology^7^.

In (3b/4a–b), we see that Greek compounds contain a so-called linking element (LE), namely, -*o*-. The LE seems similar to inflectional affixes, but Ralli (2013a) argues in detail that it has no syntactic status, and it is a mere phonological reflex. In other words, the LE does not participate in the word formation process. LEs are obligatory in Greek, and when there are not inserted, this is because of two conditions: (i) when the head element of the compound begins with a vowel higher in the sonority scale than -*o*-^8^ and (ii) when the non-head member of the compound is itself an inflected word, as in (4c–d).

Romance languages have phrasal compounds, as both elements are inflected: these are left headed, see (5) from Delfitto et al. (2011), and cf. (4c); in Italian, *uomo pesca* has only interpretation, namely, a man resembling a fish.

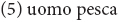



Romance languages lack LEs. Romance languages do have phrasal compounding in which a prepositional element is included within the compound very productively, as in (6), a Spanish example, from Delfitto et al. (2011):

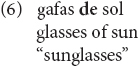



Germanic compounds are subject to the right-hand head rule (Williams, 1981) and may contain LEs (perhaps in English not as productively; see e.g., Lieber, 2009). This is illustrated in (7) with a Dutch example from Delfitto et al. (2011). Such markers are homophonous with genitive case markers or plural morphology in Germanic:

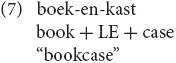



Wiese (1996) points out that since plurality may be included in English compounds, (8), such compounds involve phrases. Note that complex phrases may also be included as well as non-heads containing derivational morphology, (9), see Iordãchioaia et al. (2017) for a recent discussion; similar considerations hold for Norwegian, where left-hand members of compounds may contain derivational morphology, e.g., the derivational affix -*dom*- in *barn-dom-s-venn* “childhood friend,” see Eik (2019) for discussion and references. This is impossible in Greek (4a–b)-type compounds, e.g., ^∗^*bakal-ik-o-gatos* “grocer cat,” meaning grocer’s errand man, is ungrammatical, where the left member is derived from the stem *bakal*- containing the derivational affix -*ik*-:

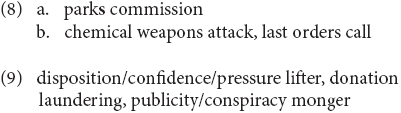



A similar point has been made by Banga et al. (2013) for Dutch. These authors report on the results of three experimental studies showing that Dutch speakers in singular contexts prefer noun–noun compounds without the LE -*en*-. By contrast, in plural contexts they show a preference for compounds with the LE. Interestingly, for the speakers participating in their studies, the LE is not just homophonous with the Dutch plural ending but it actually expresses plural meaning. This suggests that non-heads are phrasal elements.

Finally, in Turkish nominal compounds include the LE -(s)-I(n) at the right edge, (10a–b) (Kornfilt, 1997). This LE is assumed to have its origin in the third-person singular possessive agreement (Bağrıaçık and Ralli, 2015). The LE may be missing from some N–N combinations, and according to Ralli (2013b, p. 65), its absence, unlike in Greek, cannot be phonologically or structurally predicted. Bağrıaçık and Ralli (2015) argue that these compounds are phrasal, in view of the fact that plural morphology can appear on the non-head, (10b):

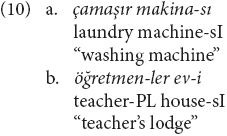



Summarizing, languages differ with respect to the presence of LEs, the position of the head, and the availability of stem-based vs. phrasal compounds. Only Greek allows stem-based compounds, i.e., the units that enter compounding in (4a–b) may correspond to bare stems, Ralli (2013a, b).

This overview leads to the following questions: what happens in cases of language contact, especially in contexts where languages do not share the same compound formation rules? Do they mix, i.e., heads and non-heads coming from distinct languages, and if so how, i.e., where do the heads as opposed to the non-heads come from? Do all language pairs mix the same way? If they do not mix, do they insert compounds from language A into the context of language B? I turn to these questions in section “Mixed Compounds in Language Contact.”

## Materials and Methods

The data reported in this paper come from a variety of sources. Specifically, the Turkish–Dutch data are taken from Backus (2003) study. Backus reports on several types of Turkish–Dutch mixing phenomena, one of which involves nominal compounds. The Dutch–French data draw from Treffers-Daller (2005), who reports on mixed compounds on the basis of two corpora: the Brussels–Dutch corpus and the Brussels–French corpus. The Turkish–Norwegian data are based on Türker (2005). The reader is referred to these publications for details.

Turning to the Greek contact varieties, the Pharasiot data are drawn from Bağrıaçık et al. (2017), who rely on various descriptive studies, and the Bovese data from Andreou (2014). The English–Greek data draw from the following sources: there are certain published studies, namely, Seaman (1972) on US Greek, and Tamis (2009) and Alvanoudi (2019) on Australian Greek^9^. When data were not available in these studies, online sources were consulted^10^, which report only on data production. The novel US–Greek data were collected as part of an experimental setting targeting language production in formal and informal settings^11^. Specifically, we collected data from speakers of Greek who qualify as heritage speakers (HS) in the sense of Rothman (2009)^12^. These speakers were recruited in New York City, NY, and Chicago, IL, in the United States. The group consists of both adults (*N* = 32, females: 16, mean age: 29.7) and adolescents (*N* = 32, females: 16, mean age: 16.2) Greek HS. In the informal part, a “chitchat” guided by the elicitor took place in order to create a relaxed and pleasant atmosphere. These were informal conversations on a variety of topics, e.g., food, films, and holidays.

In the narration task, participants were shown a short video of a fictional incident (total duration 00:42 min). They each then narrated the incident in two different communication situations, one informal to a close friend and one formal to the police in the form of a witness testimony. Within every communicative situation, the participants were asked to narrate the incident in two different modes, namely, oral and written. In the first case, they were asked to imagine that they were present in the place of the incident and they had witnessed what happened. The oral mode of the narration was to leave a message in the answering machine of the police station and the written one to provide a report typed in the laptop. During the informal setting, the participants had to narrate again the same incident to a close friend both as a text and as a voice message. Participants, after watching the video involving a car crash, had to narrate in Greek as well as in the majority language what happened. The mixed compounds were produced by speakers both in the chitchat part of the elicitation and in the narration part in the Greek part of the testing. The current size of the corpora is as follows: chitchat corpus adolescents: 29837 tokens, chitchat corpus adults: 46894 tokens, narration adolescents: 10421 tokens, and narration adults: 10349 tokens.

We note a difference in production of compounds and other switches between the narration task and the chitchat. In the former, the switches we observe involve cases of noun insertions, e.g., *accident*, *crash*, and *brake*, and of un-adapted compounds. These compounds are not equally distributed, the compound *parking lot* occurs both in the adult HS corpus and in the adolescent HS corpus, while the compound *smart car* occurs only in the adult HS corpus. Both adults and adolescent speakers produce instead of compounds mixed elements derived via affixation, e.g., *grose/ar-ia* “grocery store.” There seems to be no correlation between modality (oral vs. written) and formality. In the chitchat, however, we observe in addition discourse markers, such as *you know*, and *like*, but also mixed compounds of the type discussed in other literature.

## Mixed Compounds in Language Contact

### Turkish–Dutch and Turkish–Norwegian Mixed Compounds

Backus (2003) discusses two types of compounds in Turkish–Dutch language mixing: Dutch-type compounds, which almost never include the compound marker, (11a), and four instances of mixed compounds, which all obligatorily include the Turkish compound marker, as illustrated in (11b):

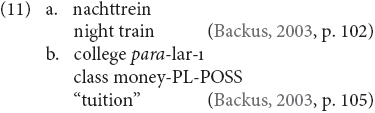



Backus argues that the mixed compounds are treated as Turkish compounds. Backus further points out that in mixed compounds the head of the compound comes from Turkish, which would then explain why the grammar treats such elements as Turkish compounds. As is shown in (11b), the LE placement follows the rules of Turkish compound formation. With respect to (11a), however, the conclusion is that these are inserted Dutch compounds. Crucially, these are compounds formed on the basis of the Dutch grammar. As has been reported in the literature and stated in the previous section – (see also e.g., Banga et al., 2013) – some compounds in Dutch lack LEs and others may appear both with and without an LE. Example (11a) would be a compound formed without an LE. Mixed compounds look like (11b): rarely if ever do we find compounds where the first element is Turkish^13^. In terms of Muysken’s typology (11b), and unlike the German–English mixed compounds discussed in Clyne (1967), it cannot be a case of congruent lexicalization, as the two languages do not share the same structure: recall that although both languages allow phrasal non-heads, the LE appears on the right in Turkish and on the left in Germanic. It appears that we are dealing with so-called insertion in this case, where elements realize a Turkish compound structure; see section “Compound Structures” for further discussion on this point.

Similar patterns are reported by Türker (2005) for Turkish–Norwegian compounds. However, unlike what has been described by Backus, Türker (2005, p. 470) reports 14 examples of Norwegian compounds used with the Turkish LE, e.g., *SAFTFLASKE****-****si-* “juice-bottle-LE,” a fact she takes as evidence that Turkish is the “matrix” language in the production of her speakers. In this case, we see that that structure of the compound is really the Turkish one, but the elements participating in the compound may both be Norwegian.

### French–Dutch Mixed Compounds

Treffers-Daller (2005) discusses several types of compounds in Brussels Dutch. Of particular interest is the case of mixed N–N compounds in her data. According to Treffers-Daller, these compounds can be divided in three groups: the first group contains compounds with a French non-head and a Dutch head, such as (12a). This is actually the largest group; the second group contains compounds with a Dutch non-head and a French head, such as (12b). Finally, the third group consists of a French non-head and a French head (12c):

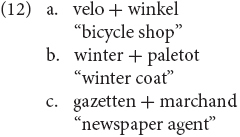



Treffers-Daller points out that in these examples the word order conforms to the Dutch grammar, i.e., all compounds are right headed. Moreover, in some cases a linking element is found:

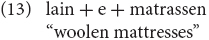



Treffers-Daller argues that the mixed compounds are best analyzed seen as instances of *insertional code-mixing*. We noted that the compounding rules differ greatly in French and Dutch. Because of this, an analysis of the above examples as *congruent lexicalization*, as put forth in Muysken (2000, p. 150), is not possible: French and Dutch do not have a shared structure for compounds. Treffers-Daller thus concludes that the French elements are embedded into a Dutch compound structure. Treffers-Daller further points out that there are also cases of borrowed compounds such as *presse-casserole* “pressure cooker,” which are listed in dictionaries. She also notes insertion of nominal groups without determiners such as *sens unique*, “one way street,” where the internal structure is French. These may often be listed in dictionaries. This suggests that the rules of the French grammar are also active.

These examples as well as the mixed Turkish–Dutch and Turkish–Norwegian examples seen above show that a structure which belongs to the one language can be filled with materials taken from two different languages. Assuming that Turkish, Norwegian, Dutch, and French compounding involves phrases, it looks like in this case words are borrowed, and compounding conforms to the rules of Dutch and Turkish, respectively. Since speakers may also produce what Backus and Treffers-Daller call un-adapted compounds, we must conclude that they have two ways of forming compounds: the un-adapted ones are part of e.g., the Dutch or French grammar. In the case of mixed compounds, however, one of the languages provides the underlying structure, i.e., is the matrix language.

### Mixed Compounds Involving Greek

There is not much work on compounding involving Greek in language contact situations. Bağrıaçık et al. (2017) discuss compounding in Pharasiot Greek, an endangered Greek Asia Minor dialect that has been heavily influenced by Turkish. The authors point out that this variety lacks typically Greek compounds, which, as mentioned in section “Rules of Nominal Compound Formation Across Languages,” are stem-based, and allows genitives as non-heads, as shown in (14). Note that (14) is not strictly speaking a case of a mixed compound, as both elements come from Greek:

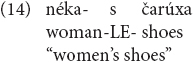



They conclude that such compounds are actually copied into the dialect from Turkish, as the canonical word order in Greek would have been as in (4d), i.e., the non-head should follow the head noun. The puzzling property is the presence of genitive case on the non-head, -s-. Bağrıaçık et al. (2017) analyze the genitive marker as an LE, which, like in Standard Modern Greek, attaches to the non-head. The arguments they provide that (14) involves a stem non-head and not a phrase include the following. First of all, they point out that the genitive in (14) appears bare, while the article is obligatory with genitives which are interpreted as possessors. Secondly, the bare genitive is non-referential. As these two properties characterize N-Ngen compounds in Standard Modern Greek, (4d), as well, they are not decisive for the stem status of the non-head. However, as Bağrıaçık et al. point out (2017, p. 198–199), evidence that the genitive marker in (14) is an LE comes from a group of masculine nouns such as the one in (15a). These appear with the -*u* suffix only when they are in the non-head position of a compound. When they appear in a genitive phrase, they bear zero marking (15b):

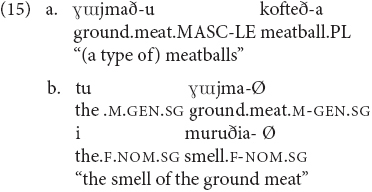



Since these nouns show stem allomorphy as other stem non-heads do in Standard Modern Greek, the authors conclude that the non-head is still a stem in Pharasiot, although the structure it is copied from involves a phrasal element. In this case, we are dealing with an interesting case of re-analysis, in which phrasal elements, i.e., words, are reanalyzed as stems, as we have seen in (1–2).

A second and clear case of mixed compounds is discussed in Andreou (2014), who studied Bovese, a Greek contact variety in Southern Italy. Andreou observes that there are no Italian un-adapted compounds in Bovese, unlike what we saw in the other contact varieties in section “Turkish-Dutch and Turkish-Norwegian Mixed Compounds” and section “French-Dutch Mixed Compounds.” What he found were examples of mixed compounds as in (16), from Andreou (2014, p. 138). In this case, either a Romance non-head is re-analyzed as a stem to enter Greek compounding (16b) or a Romance element undergoes word internal mixing to become a head in (16a):

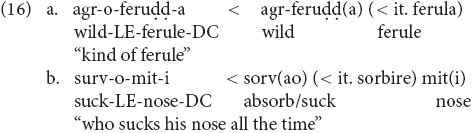



Importantly, Andreou notes that we do not have mixed compounds that show Italian headedness; this is even the case with mixed compounds such as (16a), where the head is borrowed from Romance.

These two varieties present cases of long and extensive language contact. There are also more recent examples of mixed compounds coming from Greek–English contact varieties. Seaman (1972) offers a discussion of Greek–English contact in the US. In his discussion, among other things, Seaman gives examples of nominal compounds and notes the following: first, there are nearly 200 compounds that occur in otherwise Greek environments and they occur as un-adapted forms, similarly to what we have seen in the other varieties. In (17), I include some of his examples, from Seaman (1972, p. 188):

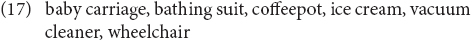



Second, Seaman (1972, p. 196–199) provides a list of what he calls adapted loanwords, and several of these involve cases of elements that are actually compounds in English but are borrowed as language internal mixes of the type in (1) and (2):

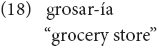



Third, he gives examples of English-Greek mixed compounds that look as in (19):

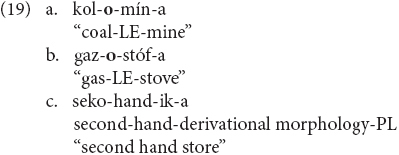



These are partially similar to (16a/16b), and, as (16a) and (16b), they involve the presence of a Greek LE. (20) offers more such examples:

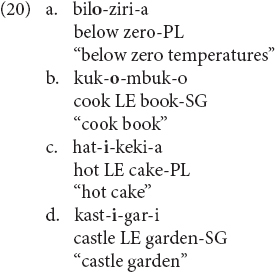



In the novel data we collected (see section “Materials and Methods”), we also found the two other types that Seaman described in his work, see (21):

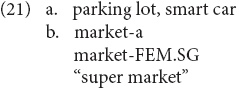



Alvanoudi (2019, p. 63) reports also un-adapted compounds for Australian Greek, (22a); see also Gardner-Chloros (2009) for British English Cypriot Greek. In this variety, we also find examples as the ones in (22b) and (22c)^14^, which are similar to the US English Greek data; see also Tamis (2009):

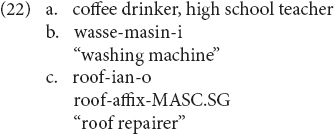



As can be seen in the examples in (19), (20), and (22b), the compounds may contain the Greek LE in addition to Greek nominal inflection on the head of the compound, as discussed in section “Rules of Nominal Compound Formation Across Languages^15^.” The latter property has been discussed in Alexiadou (2011a) and Alexiadou and Lohndal (2018), who took this as evidence for mixed word formation. In (19–20, 22b), the compounds follow the Greek compound formation rules. The presence of an LE, however, is suggestive that the speakers combine stems with the head of the compound and not phrases. Moreover, in (22b), derivational morphology, *ing*, is dropped, and the compound conforms to the bare stem constraint formulated in Ralli (2013a, b). As in Greek words, compounds are formed on the basis of stems; it does not seem that our speakers employ congruent lexicalization. These compounds are of the type in (4b), i.e., stem–word compounds in Ralli’s terms. Theoretically, they could also belong to type (4a). As, however, some of these heads occur as independent words, I will classify them as type (4b) compounds. Note that while -o- is predominantly used as an LE, in the other cases where we have reduction of ending in the absence of an LE and in some cases, -i- appears too. Recall that this element was in competition with -o- in Greek diachrony, as reported in Ralli (2013b) work (see footnote 8).

The examples in (18), (21b), and (19c/22c) provide evidence for the view in Kroll and Stewart (1994), according to which languages share underlying concepts. The concept is lexicalized via a compound in English, but with a derived word in Greek, analogically to the Greek word for this concept. Specifically, in (18) the Greek counterpart word would be *bakal-ik-o_*DC*_* “grocery store” derived from the noun *bakal-is_*DC*_* “grocer” and the addition of the affix -*ik*-; see also section “Rules of Nominal Compound Formation Across Languages.” In these English Greek varieties, speakers follow the Greek pattern and create a derived word out of the first element of the English compound.

Examples (19c/20a) require special attention: (19c) combines both compounding and derivation and (20a) is not a compound in English, it is a preposition that combines with a noun^16^. In the former case, the ending -*nd* is dropped in second and the speakers use the first element of the compound, which is itself complex, and derive a new word, adding, -*ik*-, which ends in plural. In the latter case, speakers create a novel compound, meaning below zero temperatures, and add plural morphology to the head. In (19c), this leads to the creation of a plural noun that corresponds to the Greek word for shops that sell second-hand clothes, *paliatzidika*. Typically, the use of plural on nouns referring to shops denotes areas where more than one shop is to be found. I n (20a), this leads to the creation of a so-called pluralia tantum noun, meaning long period of temperatures below zero. In fact, Greek is a language that productively has so-called plural mass nouns in Greek (see e.g., Tsoulas, 2006; Alexiadou, 2011a): these nouns bear plural morphology as count nouns, and in the presence of plurality they do not receive the container or quantity reading as is typical with mass nouns. They agree in number with the verb and cannot be combined with numerals:

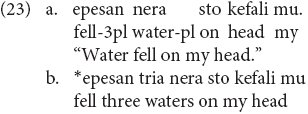



The interpretation of such nouns is, e.g., “a lot of water,” the so-called plural of abundance. Alexiadou (2011b) has argued that the distribution of plural on mass nouns and the creation of lexemes such as in (19c) in Greek resembles irregular derivational morphology; thus, plural on mass is a type of plural that creates a new category and thus it is lexical, part of the word formation process. Thus, our Heritage speakers not only create novel compounds but also novel plural nouns that conform to the properties of the Greek grammar.

To conclude this section, English–Greek contact varieties have two ways of forming compounds: they either break down phrasal English compounds and re-analyze their units or they insert the compounds as such, e.g., *parking lot*. The re-analysis comes in two shapes: either they re-analyze English phrasal non-heads as stems in order to conform to the Greek bare stem constraint or they create new words via derivation on the basis of the English non-head, and not compounding, e.g., *grose/aria*. This is a type that we did not encounter in the other language mixing pairs. Moreover, the Greek mixing cases are slightly different form the other ones, as the head is adapted to Greek morpho-syntax, i.e., it is a Greek word and not an English one. In turn, this means that speakers combine elements using the tools available to them in the system that determines the language of the compound, English and Greek, respectively. We do not seem to have cases of compounds where the one element is a fully inflected Greek word and the other an English/Romance word, i.e., mixed compounds following English/Romance compound rules or Greek rules in (4c–d) where the second element is an English/Romance word. I will come back to this in section “Units and Structures of Mixed Compounds.”

### Summary of Mixed Compounds

Before I proceed to an analysis, let me summarize the empirical picture. We have seen that in cases of language contact speakers may form mixed compounds, i.e., compounds containing heads and non-heads from two different languages as well as compounds, which are un-adapted. The Greek examples where particularly interesting as the type of mixing they contain involves re-analysis of the compound as well as creation of word internal mixing patterns.

In the contact varieties involving Greek, the compound structure is that of Greek. Greek compounds obey the bare stem constraint formulated in Ralli (2013a, b), meaning that non-heads must be bare stems. In cases of contact or borrowing of phrasal non-heads, Greek speakers re-analyze, i.e., decompose, the phrases into stems, leaving out all inflectional and other information, if the language had such type of information. (22b) is a case in point. When they come to realize then the non-head part of a compound structure, in principle they have two options: to include a Greek stem or an English stem. Both are now treated as equal from the system meaning that they must come from the same pool.

This is an important difference between Greek and the other language pairs that have been discussed in the literature. The other languages all have complex words as non-heads and not bare stems. Treffers-Daller’s data show the same type of flexibility in mixing, i.e., the system picks heads and non-heads from a unified lexicon. In the Greek mixing cases, English and Greek stems are treated on a par, the condition being that they have to appear with the linking element, obeying Ralli’s conditions, as they realize a Greek compound structure. In Dutch mixing varieties, the structure they realize is a Dutch structure, and thus phrasal elements of both languages can be inserted interchangeably. The presence of an LE is not obligatory, as is the case in Dutch. This state of affairs supports the view that the bilingual lexicon is integrated (e.g., Brysbaert, 1998; van Heuven et al., 1998; Putnam et al., 2018).

Recall that Backus, Treffers-Daller, and Türker all make a distinction between compounds lacking the LE of the “matrix” language and those that contain it. They all analyze the former as being inserted as such. We have also seen such cases for Greek. Such compounds are compounds that obey the rules of Dutch, English, and Norwegian grammar only, i.e., phrasal compounds with no Greek/Turkish LEs. Thus, bilingual speakers may resort to applying the grammatical rules of one of the two languages only.

All mixed compounds have heads that belong to one language only. Turkish–Norwegian is here the exception, as SAFTFLASKE-si- “juice-bottle-LE” is possible. While both Turkish and Norwegian are right headed, the compounds are LE final, even if the head is Norwegian and thus they conform to the Turkish compound rules. The French–Dutch mixed compounds follow the rules of Dutch compound formation. The mixed compounds in the English Greek contact varieties have Greek headedness.

In all cases, we have what Myers-Scotton (1993) labels a matrix language that determines the morpho-syntactic frame of the compound. In principle, this matrix language could be determined by sociolinguistic factors and/or by grammaticized features; see the discussion in section “Introduction.” The clearest case where the latter is at work is mixed Greek compounds which have grammaticalized LEs. Turkish–Dutch and Turkish–Norwegian could also be cases of this since Turkish, as Ralli (2013b) argues, uses LEs to indicate the morphological structure of compounding^17^.

## Units and Structures of Mixed Compounds

### Theoretical Premises

In this section, I will introduce some basis of the framework I adopt to explain the above generalizations. Work within the framework of distributed morphology is based on the idea that all words are complex, i.e., multimorphemic. Word formation involves the combination of acategorial roots with functional elements, as illustrated in (24) (see Marantz, 2007; Embick, 2010) for overviews. n and v are so-called categorizing heads, creating nouns and verbs. These heads are associated with e.g., inflectional class and gender features in the case of nouns in languages that have such features or event implications in the case of verbs.

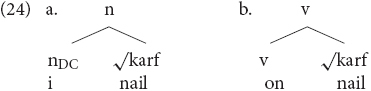



Every language has a set of roots and a set of vocabulary items that spell out functional categories such as n, i.e., DC information, and v and other functional categories such as Tense and Aspect, which have to be language specific. From this perspective, every word is complex. In (24), the Greek root √karf “nail” can combine either a nominal head bearing inflectional information as in (24a) or with a verbal head realized via -on-^18^.

The data illustrated in (1–2) can thus be accounted as follows: Alexiadou (2011a) argued that what in these examples English and German roots combine with a Greek nominal head which, following all typical features of Greek nouns, will realize declensional information. This is illustrated in (25), the morphological structure of the example in (1):

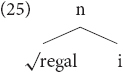



Alexiadou and Lohndal (2018) note that since word internal mixing is asymmetric, i.e., in Greek–English, Greek determines inflection, while in Spanish–German, German is the language of inflectional information, this preference is guided by overt realization, i.e., the language that provides overt realization for a particular head is the one chosen by the bilingual speakers, e.g., Greek in the case of German–Greek contact, but German in the case of Spanish–German contact, and cf. Muysken (2000) for additional factors.

### Compound Structures

Assuming the model in (24), Iordãchioaia et al. (2017) argued that Greek compounds involve stem non-heads, while English compounds phrasal non-heads. Simplifying their analysis quite a bit, we can assume the structures in (26)^19^. (26a) is the structure for examples such as (4b) and (19b), which are relevant here for our mixed patterns, as the second element of mixed Greek compounds is a word that contains internal mixing. I assume, following Ralli (2013b), that the LE is inserted at morpho-phonological structure and is not part of the morpho-syntax of the compound. (26b) corresponds to an English compound structure:^20^

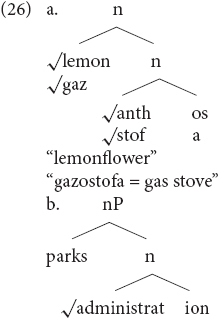



In the Greek mixing varieties, Greek speakers make use of (26a), in which case a mixed compound will appear as having an English-based stem as a non-head combining with a head which is itself the product of the structure in (25). This is the reason why pattern (4a) is not found: the English stem must become a Greek word to enter Greek compounding. They may also make use of structure (26b), in which case they produce un-adapted compounds such as *parking lot.*

The question that arises is why Greek speakers necessarily re-analyze, in other words what would block (4c–d) type of structures. Theoretically, (4c–d) could be possible, but then both elements would need to first undergo a process of word internal mixing creating words of the type seen above in (1–2). Such examples, which are not attested, would look like in (27a–b), theoretical examples partially constructed on the basis of individual nominal forms found in Seaman (1972) study, where the forms that have undergone word internal mixing are underlined:

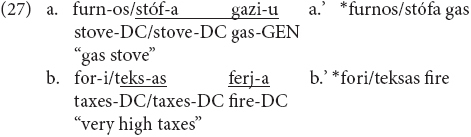



Recall that pattern (4d) is a case of word–word compound in Greek, in which the non-head bears genitive. Thus, if an English non-head would be part of the compound it would like (27a) and not like (27a’). Moreover, (4c) also involves fully inflected forms, which again makes it less likely for the non-head to come from English without any word internal mixing, thus (27b) and not (27b’).

The situation is different in the other pairs discussed in this paper, where compounding is phrasal. While also these speakers have access to a monolingual structure of the type in (26b), they have other options to realize it: in the case of Dutch and French, speakers basically again realize a structure of the type in (26b) with materials either from French or from Dutch; in the case of Turkish–Dutch, Turkish–Norwegian mixed compounds, again, we have a basic Turkish structure and speakers realize the structure by taking elements from both their languages.

Finally, the concepts that the structures in (26) express can be lexicalized either by a compound or by a derivational process in Greek, e.g., (18). It remains to be investigated if derivation instead of compounding is a pattern only characterizing mixing varieties that involve Greek, where compound formation is subject to the bare stem constraint.

## Conclusion

In this paper, I discussed cases of compound formation in language contact situations. This discussion is informative for the status of bilingual grammars and the resources bilingual speakers have at their disposal. Importantly, I showed that speakers may also re-analyze the constituents of compounds, i.e., make use of stems instead of phrases, if the rules of their two languages are in conflict with respect to the size of the compound constituents. This is clearly the case in mixing involving Greek. The study of compounds in language contact informs about the units of mixing as well as of the organization of the mental lexicon of bilingual speakers. Specifically, we find evidence for Kroll and Stewart (1994), according to which languages share underlying concepts but lexicalize them in different ways. Moreover, we have seen evidence for the view that the bilingual lexicon is integrated, as our speakers can pick from both languages the materials to realize compound structures (e.g., Brysbaert, 1998; van Heuven et al., 1998; Putnam et al., 2018), irrespectively of the question of its internal structure (see Alexiadou and Lohndal, 2018; López, 2018). The creativity of bilingual speakers in the formation of compounds in the case of mixing shows that they may pick phrases but also units smaller than phrases from one language and introduce in a grammatical structure of another. Irrespectively of how exactly this can be modeled, it provides further evidence for the internal complexity of words and their decomposition as well as the gradience between compounding and derivation.

Moreover, the fact that speakers can pick units smaller than words/phrases has implications for experimental work on compound processing as well as derivational processing, an issue that has been discussed controversially in the L1 but also L2 literature. In a recent review article on morphological processing in the brain, Leminen et al. (2019) discuss neuroimaging literature on inflection, derivation, and compounding. The authors point out the following, Leminen et al. (2019, p. 37): “The picture offered by the review of the studies investigating derivational morphology is much hazier (and hence the ‘bad’ in the title) than the review of inflectional morphology. Most of the studies suggest that the activation and response patterns support decompositional, two-stage (orthographic and semantic) or dual-route accounts, but the latency of morphological effects as well as their localization differ greatly depending on the paradigm and linguistic variables,” see also Silva and Clahsen (2008) for L2 derivational morphology. Importantly, however, they state that “the short review of the few studies exploring compound word processing demonstrates that this is one of the key morphological operations that requires further attention and that needs to be developed given the scarcity and volatility of the results (and hence the ‘ugly’ in the title). While some studies clearly support views favoring the access to the constituent morphemes prior to accessing the whole compound word, some other neuroimaging studies posit that compounds are processed at a whole-word level. Moreover, while some studies suggest that the semantic transparency of compound words may determine the manner in which these words are accessed, others claim that transparent and opaque compounds are processed similarly. Furthermore, there are studies suggesting that the extent to which constituents can be accessed highly depends on the prior experience with the whole compound, claiming for differences in the morpho- logical decomposition of novel and existing compounds.” Thus, bilingual mixed as well as un-adapted compounds provide a fruitful area to further test and elaborate processing accounts.

Naturally a series of questions emerge^21^. A first question is: do bilingual speakers make productive use of the compound rules described in this paper? To answer this satisfactorily, experimental research with novel compounds is necessary. Our data as well as the other data on Greek reported here are production data so we do not know what speakers would do in the case of novel compounds. A second question is what determines whether they use an un-adapted compound or a mixed one? In principle, a variety of reasons could play a role. It could very well be that this is proficiency related, i.e., more proficient speakers use borrowed compounds. Gardner-Chloros (2009) points to this direction in her work on British English Cypriot Greek language contact situation in London saying that borrowed compounds characterize the production of balanced bilinguals. I mentioned in footnote 15 that preliminary results from German Heritage speakers suggest that they primarily make use of borrowed compounds. If this is indeed the case, we can speculate that the mixed compounds found in the US Greek Heritage speakers’ production remain in the grammar as forms created by the 1st-generation immigrants, who were not balanced bilinguals (Seaman, 1972). The study of Greek contact varieties that have been in a language contact situation for a long period of time, e.g., Bovese, suggests that unadapted compounds are not used, as Bovese has developed its own compounding mixed system on the basis of Greek. Alternatively, it could be that different communities adopt different conventions, and the reasons for that need to be clarified. It could also be the case that the typology of compound formation plays a role. Greek is different from the other languages in that it builds stem-based compounds. However, the stem as opposed to phrasal nature of compounds did not seem to affect the existence of mixed compounds. What I did observe, however, is that mixing varieties that involve Greek may use derivation instead of compounding, e.g., (18) and (22c). This might indeed be related to the morphological parameter in compounding. Finally, I focused on cases in which Greek is the matrix language. Are there mixing varieties where Greek is not the matrix language and how does compounding work in these? Mileva (2009) reports on code switching between Greek and Bulgarian by recent Bulgarian immigrants in Northern Greece. In her data, we find Greek compounds of the type (4d) as well as (4b), e.g., *ársi varón* “lift-weights-GEN” and *spit-o-nikokirá* “house-LE-lady” in an otherwise Bulgarian frame. Mileva characterizes her speakers as showing a high degree of bilingualism suggesting, as mentioned above, that indeed proficient speakers use un-adapted compounds. All these issues await further research.

## Data Availability Statement

The datasets generated for this study are available on request to the corresponding author.

## Ethics Statement

The studies involving human participants were reviewed and approved by Deutsche Gesellschaft für Sprachwissenschaft. Written informed consent to participate in this study was provided by the participants and by the participants’ legal guardian/next of kin in the case of the adolescent participants.

## Author Contributions

The author confirms being the sole contributor of this work and has approved it for publication.

## Conflict of Interest

The author declares that the research was conducted in the absence of any commercial or financial relationships that could be construed as a potential conflict of interest.

## Footnotes

1 I use here the label language mixing to refer to patterns where elements of two languages are combined. As correctly pointed out by a reviewer and is duly acknowledged in Alexiadou and Lohndal (2018) as well, one can really only talk about preferences rather than categorical (im-)possibilities relying on judgments, since such judgments are often negative due to sociolinguistic reasons. For this reason, the data reported in section “Mixed Compounds in Language Contact” are drawn from corpus evidence and do not rely on introspection.

2 The main point here is that while what we see is a type of insertion (cf. Muysken, 2000), what is inserted is an element that is smaller than a fully inflected word. As Alexiadou et al. (2015) note, not all DCs are equally available for selection; see also Ralli et al. (2015). In Greek, determiners agree in gender with the noun they are associated with. Alexiadou and Lohndal (2018) point out that the gender determination in these mixes is either analogical, i.e., the noun receives the gender its Greek counterpart also has, or a gender that corresponds to the default gender, namely, neuter. In some cases, the noun receives a gender that does not correspond to the gender of the Greek or the German noun, e.g., *i kel-a* “the cellar-FEM.SG” vs. *to kelar-i* “the cellar-NEUT.SG” and *der Keller* “the-MASC.SG cellar” (see Alexiadou, 2011b; Alexiadou et al., 2015) for details on the German data, and see also the discussion in Alvanoudi (2019) for English–Greek mixing in Australia. A reviewer asks how the separation between stem and affixes in the word internal mixing was determined. In, e.g., (1) and (2) the German and English words appear with a Greek inflectional affix, thus in these cases they function as stems. In the cases we will see in section “Mixed Compounds in Language Contact,” e.g., *grose/ar-ía* “grocery store,” the separation assumed that the English stem is *grocer* and affixes attach to it, which bear Greek stress. In addition, this stem appears in two forms *groser* as well as *grosar*, suggesting stem re-adjustment on the basis of affixation. Moreover, speakers also produced the form *grosar-ik-a* “vegetables,” where a derivational affix, -*ik*- and a plural inflectional affix -*a-* are attached. In general, when possible, the DC rules provided in Ralli (2000) have been followed.

3 As a reviewer points out, this suggests that societal factors are involved: the community language sets the syntactic frame, while the societally dominant language is the lexifier.

4 Note that, according to Muysken (2000, p. 3), congruent lexicalization differs from insertion: the former involves congruent lexicalization of material from different lexical inventories into a shared grammatical structure, while the latter involves insertion of material (lexical items or entire constituents) from one language into a structure from the other language. Treffers-Daller (2005) argues that certain mixed French–Dutch compounds are cases of insertion, as we will see below.

5 Moreover, again following Ralli (2013a), I will not consider as compounds so-called lexicalized phrases such as the expression *forget me not*.

6 A clarification is in order: in this section, I use the term stem for Greek compounding. In morphological theory, however, there is a distinction between roots and stems. Root is defined as the part of a word that cannot be further analyzed, i.e., the form without any inflectional or derivational affixes. By contrast, one speaks of stems in the context of inflectional morphology (see, e.g., Bauer, 1983). For example, in English examples such as *teapot* are considered root compounds, as they are taken to contain two roots, but see below. The element *teapot* is also a stem as inflectional morphology can attach to it. With respect to Greek in particular, the literature largely follows Ralli (2013b) in adopting the view that there is no distinction between roots and stems and that stem is the basic word formation unit in the language. Ralli (2013b, p. 8) states: “in Modern Greek, the borders between stems and roots have been blurred, and there is no syntactically motivated distinction between the two. However, the situation was different in Ancient Greek, where a stem was basically a combination of a root and a thematic vowel. Today, there are not structural boundaries between a root and a thematic vowel.” Following this tradition, I will refer to Greek as a stem-based language for the purposes of compounding, while all other languages build compounds on the basis of phrases/words, i.e., stems or roots plus additional morphological information (inflectional or derivational); see (8–9) for English.

7 In Greek, all type of compounds, irrespectively of their internal complexity, function as names for, e.g., entities, see Ralli (2013b) and Gavriilidou (2013) for further discussion.

8 As can be seen in the examples in (3) and (4a–b), the LE is not part of the inflectional ending, as the words in isolation would have different endings. The sonority and stress hierarchy discussed in Ralli (2013b, p. 17) is shown in (i):

(i) á > a > é > e > ó > o > í > i > ú > u

Ralli (2013b) also points out that a further case in which the LE is missing is with stems of Ancient Greek origin. There are some compounds that have the LE - i-, e.g., *lem-i-tómos* “neck cutter,” which are also of Ancient Greek origin. As Ralli (op.cit.) reports, in Modern Greek the LE -o- prevailed over the LE -i-.

9 I am indebted to an reviewer for pointing out to me the existence of this study. Seaman recorded forty-one individuals, most of which lived in the greater Chicago area. The informants were 1st- (13), 2nd- (20), and 3rd- (5) generation immigrants; he places 3 in a separate category, 2–3 generation. Seaman’s classification is as follows (Seaman, 1972, p. 21): 1st generation = born in Greece and immigrated to the United States; 2nd generation = born in the United States of 1st-generation parents; 3rd generation = born in the United States of 2nd-generation parents; 2nd–3rd generation = born in the United States of one 1st-generation parent and one 2nd-generation parent.

10 https://usa.greekreporter.com/2019/11/20/greeklish-the-linguistic-creativity-of-greek-americans/ and https://au.greekreporter.com/2016/08/19/to-booko-greek-australian-dictionary-of-the-greekish-dialect/

11 The data collection was carried out within the frame of the project AL 554/13-1 *Nominal morpho-syntax and word in Heritage Greek across majority languages* (Project number 394836232), part of the Research Unit 2537 *Emerging grammar.* The narration corpus is available online, https://zenodo.org/record/3236069#.XnoI1C1oTKI. We are in the process of gathering comparable data from Greek Heritage Speakers in Germany. Thus far, we have collected data from 27 adults (females: 17, mean age: 28,4) and 21 adolescents (female: 7, mean age: 16, 5). The approximate total number of tokens of both US Greek HS and Germany Greek HS production is currently at 71,000 tokens.

12 According to Rothman (2009, 156), “a language qualifies as a heritage language, if it is a language that is spoken at home or otherwise readily available to young children, but, crucially, this language is not a dominant language of the larger (national) society.” The speakers that participated in our study qualify as such on the basis of this definition.

13 A reviewer points out that this must be related to a specificity principle: compound nouns have the semantic structure of a modifier modifying a general type of thing (the head noun). Since basic vocabulary is rarely borrowed, this predicts compounds of the type in (11b). This would also explain why in the Dutch–French mixing variety discussed in section “French-Dutch Mixed Compounds,” the majority of compounds are as in (12a).

14 (22b–c) come from https://au.greekreporter.com/2016/08/19/to-booko-greek-australian-dictionary-of-the-greekish-dialect/.

15 Looking at the Greek–German variety in our corpus, at first sight these speakers seem to be using un-adapted/borrowed German compounds, which we might want to attribute to the fact that the German speakers are more balanced bilinguals; see the remarks on Gardner-Chloros (2009) study in the main text. This awaits further investigation.

16 A reviewer points out that the presence of the linking element is not immediately clear in this example as it ends in an open syllable. According to Ralli (2013b) rules, in this case the LE would be left out obligatorily for morpho-phonological reasons.

17 Note here that this is in agreement with much work in sociolinguistics on the asymmetry of such relationships and the ideas about basic vocabulary and its provenance; see footnote 13.

18 A reviewer asks whether there are cases of Greek nominal affixes attached to items that are clearly verbs in English. The cases I could find in the literature and our data seem to involve roots that could become both verbal and nominal as in (24). Tamis (2009) reports that in Australian Greek 35% of the English transfers are from the nominal domain, as they denote concepts of occupation, home environment, and place of work. Theoretically, it would be possible to simply take any English root and create a noun out of it, an option available to the monolingual grammar as well. If bilinguals, however, want to create a noun out of an e.g., English verb, they would need to first create a Greek variant, i.e., make use of word internal mixing to create a verb, which then becomes a noun via affixation, since the morpho-syntactic structure is determined by Greek. A Greek nominalizer could not attach to an English verb as this would be allowed by Greek phonotactics; see Alexiadou and Lohndal (2018) for discussion. Seaman (1972, p. 166) reports the Greek nominal form *ena vreksi* “one rain,” a case of nominal use of a Greek verbal form, which he attributes to the influence of English, i.e., to the fact that English has several forms that could both function as verbs or nouns.

19 See Ralli (2013b) and Gavriilidou (2013) for alternative structures.

20 As mentioned, structure (4a) is in principle also theoretically possible, as in (i):

(i) [_n_ √√].

21 Many thanks to a reviewer for suggesting several possible avenues to explore.
